# Barriers and facilitators of physical activity, sedentary and sleep behaviours in 3 to 4-year-old children from low-income families: a study protocol

**DOI:** 10.1186/s44167-023-00030-8

**Published:** 2023-11-01

**Authors:** Andrew Dalziell, Xanne Janssen

**Affiliations:** Department of Psychological Sciences and Health, University of Strathclyde, 50 George Street, Glasgow, G1 1QE Scotland

**Keywords:** 24-Hour movement behaviours, Physical activity, Sleep, Screen time, Sedentary behaviour, Low-income families

## Abstract

**Background:**

This study will evaluate the barriers and facilitators that families experience in adhering to the 24-hour movement behaviours guidelines as outlined by World Health Organisation (WHO).

**Methods:**

The study is a mixed-methods study and will recruit between 20 to 30 low-income families with children aged 3- to 4-years living in Scotland. For the quantitative part, children will be asked to wear an Actigraph (GT3X +) accelerometer to measure physical activity, sedentary behaviour, and sleep. Parents/guardians will be asked to keep an activity diary outlining when their child has had to remove the device (i.e., showering, bathing, swimming) and record the child’s screen time each day. Once the data has been analysed, a unique activity profile chart will be sent out to each family illustrating their child’s 24-hour movement behaviours (i.e., time spent active, time spent sedentary and on screens, time spent sleeping). The activity profile will provide a day-by-day output as well as a weekly average for each of the 24-hour movement behaviours. Qualitative data will be collected using the Asynchronous Remote Communities method (ARC). The ARC involves participants completing activities using an online closed Facebook group. Parents/guardians of 3- to 4-year-old children will be asked to engage in group discussion tasks using the private and closed-group online platform (a minimum of 6 and a maximum of 8 families per discussion group). The quantitative data collated from the questionnaire and activity monitor will be presented through descriptive analysis and after the 6-week asynchronous process is complete, qualitative data will be collated and analysed using Braun and Clarke’s reflexive approach to thematic analysis.

**Discussion:**

The data collected will provide an understanding of what barriers and facilitators parent’s/guardians’ experience in relation to adhering to the 24-hour movement behaviour guidelines. This could potentially lead to the design and implementation of support and interventions to help families struggling to adhere to the guidelines.

## Background

The early years of a child’s life (birth to 5-years) is crucial for physical, emotional, social, and cognitive development [1, 2]. Many aspects can influence the trajectory of this development such as genetics, environment, gender, parental educational level, socio-economic status (SES), and lifestyle behaviours such as physical activity, sedentary behaviour and sleep [3]. Not all children have the same life opportunities with population studies showing a significant disparity in healthy behaviours between low-, middle-, and high-income families [3]. Reduced access to green spaces, increased use of screens and increases in motor transportation are only a few of the factors impacting time spent in physical activity and sedentary behaviour in young children [1].

Physical activity, sedentary behaviours, and sleep play a key role in the health and development of young children and recently several countries around the world and World Health Organisation (WHO) released guidelines on physical activity, sedentary behaviour, and sleep for children under five-years of age. The guidelines indicate that children aged 3- to 4-years should engage in at least 180-min of physical activity including 60-min of moderate-to-vigorous physical activity (MVPA), no more than one-hour of sedentary screen time (SST), and 10- to 13-h of quality sleep to build a better day [4]. Following the release of the guidelines several studies from across the world have shown most young children are not meeting these guidelines [3, 5, 6]. For example, a recent international cohort study, including 14 countries, showed the prevalence of meeting all three guidelines was 14% before the COVID-19 pandemic and this number decreased even further during the pandemic (11%) [7]. It is clear from these latest figures that to build a better day and empower families to opt for a healthier lifestyle, targeted interventions are needed. Yet, targeting each of the 24-hour movement behaviours individually is not effective to fully address the situation. It is important to recognise that these movement behaviours interact with each other, a change in the time spent in one behaviour will consequently result in a change in the time spent in at least one of the other behaviours. For example, time spent being sedentary (including screen time) increase with age and displaces time spent in physical activity or sleep [8, 9].

We must acknowledge that not enough is known about the factors influencing multiple health behaviours in the early years [10]. Much of the previous research has provided some insights into the barriers and facilitators for building a better day which provides useful directions. However, many of these studies have involved the participation of middle to high-income families [2, 11–13]. It is imperative that we gain a deeper understanding of the barriers and facilitators experienced by low-income families when trying to build a better day. Yet, low-income families are often harder to reach in terms of research and therefore this current study is essential as the potential barriers and facilitators experienced by low-income families may be different.

While previous systematic reviews have highlighted a range of promising intervention settings, professionals, and components (i.e., the features that define an effective program), there is a need to gauge the acceptability of these in families living in deprivation to maximize potential for effectiveness [5, 14]. Therefore, the present study aims to understand the barriers and facilitators low-income families experience when trying to adhere to the WHO guidelines for physical activity, sedentary, and sleep behaviours of children aged 3- to 4-years-old.

The proposed research is designed to gain a better understanding of the families’ perceptions, needs and gaps to assist with building a better day and reduce the obesity inequalities in pre-schoolers among families living in deprived areas. This study will therefore draw on quantitative data to provide clear representations of the 24-hour movement behaviours. However, this in isolation will not provide an accurate account of participants actual experiences. Therefore, qualitative data will be used to hear directly from the participants about their experiences in trying to build a better day for their child (see text box below). By combining quantitative with qualitative methods, we can employ an innovative method in encouraging participants to engage in more detail in the qualitative aspect of the study. The insights gained will help to develop and improve the effectiveness of intervention targeting these behaviours. The novel methods used in this research will assist in the interaction with hard-to-reach families in Scotland and provide important insights for other researchers working with families in deprived areas. This protocol paper will clearly outline the methods adopted within this study.

## Methods

### Design and recruitment

The study will use a mixed methods approach collecting quantitative and qualitative data. The quantitative data will be used to inform the qualitative phase. We aim to recruit between 20 to 30 low-income families with children aged 3- to 4-years living in Scotland. Low-income families are defined in Scotland as earning less than 60% of the national median pay [14]. To help identify low-income families, nurseries within the lowest 1st to 3rd decile within the ‘Scottish Index of Multiple Deprivation (SIMD)’ will be asked to display a recruitment poster and to encourage their families to learn more about the project and participate. SIMD is widely accepted as a measure of deprivation. In addition, the recruitment poster will be posted on social media platforms to raise awareness and encourage further recruitment opportunities. Parents/guardians who are interested in participating will be asked to scan the QR code on the recruitment videos, posters, or to use the weblink provided. These links will take them to an online information and consent form which includes a recruitment video outlining the purpose of the research, the tasks the participants will undertake, and how their data will be managed. This online form will also include an eligibility check to ensure participating families are within the low-income category defined as earning < £16,000 per annum (i.e., 60% of the median pay in Scotland in April 2022).

### Ethics approval

Ethical approval has been obtained from the School of Psychological Sciences and Health Ethics Committee at the University of Strathclyde and informed consent will be obtained from all participants. Participants will be provided with a ‘Participant Information Sheet (PIS)’ outlining their involvement. They will then be asked to sign and date the consent form, with the understanding that they have the right to withdraw from the study at any time. Consent will be obtained through Qualtrics for participants enrolling online, and in paper format with participants who sign-up face-to-face.

### Measures

#### Demographical questionnaire

Parents/guardians will be asked to complete a brief online questionnaire, or a paper copy when conducting face-to-face recruitment. The questionnaire will include demographical questions with regards to their child’s physical activity, sedentary behaviour, screen time and sleep.

#### Physical activity, sedentary behaviour, and sleep

Physical activity, sedentary behaviour, and sleep will be measured using an Actigraph (GT3X +) accelerometer. The Actigraph is a widely used activity monitor which has shown acceptable classification accuracy for sedentary behaviour (ROC-AUC = 0.80), light physical activity (ROC-AUC = 0.66), moderate-to-vigorous physical activity (ROC-AUC = 0.70) and sleep (PABAK = 0.76) in this age group [15, 16]. Children will be asked to wear the accelerometer on their right hip continuously for at 7 days, except during water-based activities, using an elastic waist band. Parents/guardians will keep an activity diary outlining where their child has had to remove the device (i.e., showering, bathing, swimming). Actigraph data will be collected at 100 Hz. Raw data will then be downloaded using ActiLife version 6.12.1 (Actigraph, Pensacola, FL, USA) and converted to 15-s epochs. Parent/guardian reported activity diaries and manual visual screening will be used to identify non-wear periods and the start of bed and wake times. Excluding non-wear and sleep periods, daily sedentary behaviour, light physical activity, and moderate-to-vigorous physical activity will be calculated. Epochs will be defined as sedentary behaviour, light physical activity and moderate-to-vigorous physical activity when recorded counts are < 200 counts/15 s, between 200 and 799 counts/15 s and > 800 counts/15 s respectively [17]. Sleep time per day will be calculated using the Sadeh algorithm ActiLife version 6.12.1 (Actigraph, Penscola, FL, USA) [18]. This algorithm has previously been validated for use in young children [16]. The time flags for sleep onset and sleep offset will be determined based on the participants activity diary. Participants with at least 3-days of valid data will be included in the analysis. A valid day is defined as having at least 10-h per waking day of accelerometer data [19].

#### Screen time

**Fig. 1:**
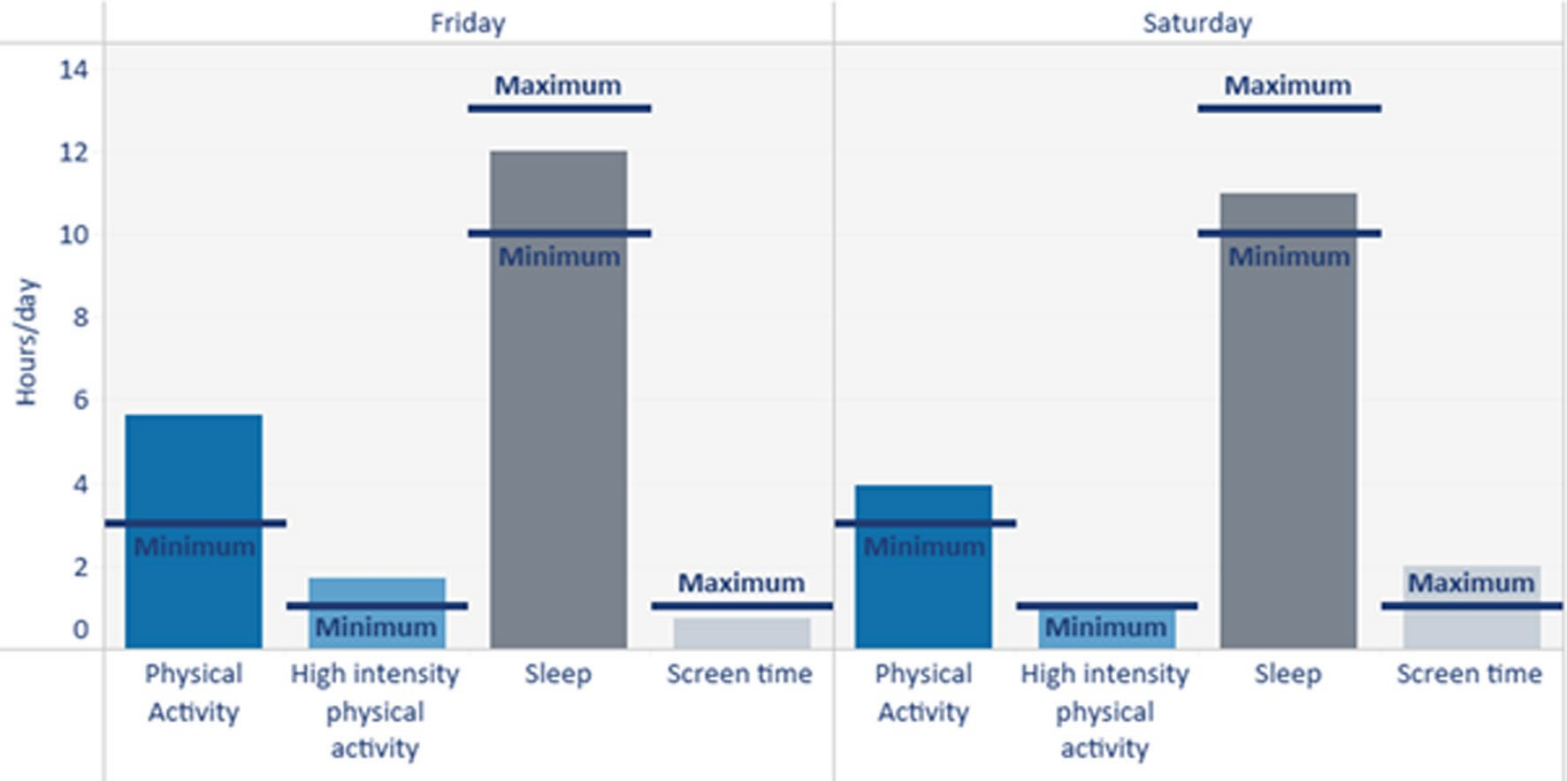
Activity profile chart

Screen time is measured using parent/guardian reports. Parents are asked to record their child’s screen time on the activity diary for each day (see Appendix 1). Once the device has been returned and the data analysed, a unique activity profile chart (see Fig. 1) will be sent out to each family illustrating their child’s 24-hour movement behaviours (i.e., time spent active, time spent sedentary and on screens, time spent sleeping). Parents/guardians will also be asked to complete a brief online questionnaire. This questionnaire will include demographical questions as well as questions with regards to their child’s physical activity, sedentary behaviour, screen time, and sleep.

#### Qualitative data

Qualitative data will be collected using the Asynchronous Remote Communities method (ARC). The ARC involves participants completing activities using an online closed Facebook group. This method is recommended for seldom heard and hard to reach populations as it removes barriers such as availability and difficulty of travel, often associated with face-to-face focus groups [20, 21]. Parents/guardians of 3- to 4-year-old children will be asked to engage in group discussion tasks using the private and closed-group online platform (a minimum of 6 and a maximum of 8 families per discussion group). There will be two activity posts each week for a period of 6-weeks where parents/guardians will post comments and opinions related to each of the activities designed for this study. The activities aim to gain an understanding of the barriers and facilitators parents/guardians face when building a better day and the settings and key people involved in promoting and supporting healthy behaviours in their children. The personalised activity profiles created during the quantitative phase will be central for picturing their child’s 24-h day and aims to increase engagement during the qualitative component. Where parents/guardians are not interacting with the online discussion, personal reminders will be sent by ‘tagging’ them into the post or by sending them an email reminder.

### Study protocols

Following the consent process participating families will be asked to complete a short questionnaire and will be sent out their activity monitor (Actigraph GTX3 +) by the lead researcher. The activity monitors will be mailed alongside instructions on how to attach them, who to contact if issues arise, and the activity diary to note the times the monitor is removed and to record bedtimes and wake times. Children will be asked to wear the monitors for seven consecutive days for the whole 24-h each day, in line with standardised protocols used in large studies globally [22]. During this time, they will not receive any information regarding their movement behaviours and will be asked to maintain their usual daily activities. On completion of the seven consecutive days, the monitor will be returned using the pre-paid envelope provided. If participants are failing to return the monitor, reminders will be sent out to participants. No incentives will be provided until the monitor has been sent back to the research team.

Once the monitors have been returned, participants will be sent a link to the closed online platform and their unique activity profile providing an easy-to-understand visualisation of a typical day in the life of their child’s 24-hour movement behaviours. Where there is insufficient data from the Actigraph, or the device has not been sent back, an exemplar visualisation will be provided to allow the participant to continue to engage in the qualitative aspect of this study and participants will be offered to wear another monitor. During the asynchronous process, members of the research team will post two activities each week (Textbox 1). Following a brief introduction, the activities will start with a brief discussion about 24-hour movement behaviours and to identify how useful the unique activity profiles were in understanding their child’s 24-hour movement behaviour. Further activities will then focus on parents/guardians understanding of the guidelines, key stakeholders who could help them build a better day for their child and any potential barriers and facilitators they experience. Activities will include both group and individual tasks providing opportunities for participants to express their views, opinions, and experiences. In addition, parents/guardians will be able to post pictures as part of these activities. Upon completion of all tasks within the study, participants will be thanked and sent a £15.00 voucher as a token of appreciation.

### Data management

All data collected will be in electronic form. Any files will be password protected and stored on the University online password protected storage systems. Data will be pseudo-anonymised, so any raw data is anonymised and given a code name (e.g., P001, P002 etc.) with the key for code names being stored in a separate location from the raw data. This is to ensure identifying information cannot be linked to data extracted from participants.

The extracts from the asynchronous tasks will be copied into a text document and stored on the University online password protected storage systems. Participants names will be replaced with their participant code. Once all data has been extracted from the online platform, the page will be terminated.

The lead researchers and research assistant will have access to the key for the code names. The de-identified data from this study will be made available as “open data” through a research data repository. Information such as name, address and email will not be included in the dataset uploaded. The database will remain there indefinitely. Any person who wants access to that data on the repository will need to submit a data request explaining why they want to use the data.

### Analysis

The quantitative data collated from the questionnaire and activity monitor will be presented through descriptive analysis showing medians and ranges for the movement behaviour outcomes as well as showing the percentage of children meeting the movement behaviour guidelines. After the 6-week asynchronous process is complete, qualitative data will be collated and analysed using Braun and Clarke’s reflexive approach to thematic analysis [23]. The main researcher will adopt six phases within the data analysis in line with the recommendations from Braun and Clarke. Firstly, the main researcher will familiarise themself with the data by completing the transcriptions and reading through these transcripts. Secondly, initial codes will be generated after completing the first step. Thirdly, themes will be searched for through the data collected. Fourthly, themes that have been generated during the second phase will be reviewed. Fifthly, themes from the research will be defined and named, before finally producing a report. Qualitative data will include anonymised transcripts of discussions and pictures from the ARC method obtained during the 6 weeks using a closed Facebook page. These will be analysed thematically using NVivo software (Version 1.7, 2022). The main research assistant will produce a final report which will be sent to an external researcher with experience in using the NVivo software for thematic analysis. Discussions between both researchers will determine if the themes, coding, and classifications identified is accurate and provides a detailed account of the main themes and sub-themes within the qualitative data. Where there is disagreement, a third researcher will moderate the findings. An analysis will be undertaken to identify if families who adhere to the guidelines have a different perspective to those who did not adhere to the guidelines.

### Textbox 1: Key topics covered in qualitative research components

Key qualitative topics

- Thoughts and reflections on the 24-hour movement behaviours.
- Perceptions of their child’s actual 24-hour movement behaviours.
- How do families incorporate the 24-hour movement behaviours daily.
- Do 24-hour movement behaviours affect child’s general behaviour.
- What are the main barriers to achieving the WHO recommendations.
- What helps families achieve WHO recommendations.
- What participants feel would help them build a better day.

## Discussion

Innovation is embedded in this research topic and methodology. There is a low percentage of children aged 3- to 4-years meeting the WHO guidelines [4], but very little understanding of why this is the case. This will be the first study to focus on parents’ / guardians with deprived families’ perceptions of, and barriers and facilitators to, the 24-hour movement concept in deprived communities globally. Up until now studies have focused on these behaviours individually, however, as indicated above, they all rely on each other and treating them as such is important [8, 9]. In addition, the research uses novel methods to engage a hard-to-reach community. It has been reported previously that families from deprived areas are often underrepresented in research studies and more effort is needed to reach out and include them [24]. Very little is known about barriers and facilitators of these behaviours in deprived communities. The ARC studies design has shown to be more effective in involving hard to reach populations [20, 21]. By combining quantitative with qualitative methods, we can employ an innovative method in encouraging participants to engage in more detail in the qualitative aspect of the study. By providing our participants with personalized activity profiles of their child’s 24-h day they will be able to reflect and think more clearly about what may have influenced their day, what barriers and facilitators they face and how these behaviours interact. The information collated and analysed may help to improve our understanding of the genuine barriers and facilitators that families experience when trying to build a better day for them and their child. This would provide a unique opportunity to identify influences on the adherence to the 24-hour movements guidelines in pre-schoolers from low-income families and would provide new evidence for policy makers, health practitioners and other stakeholders to design more effective interventions to target and increase health promotion efforts and early intervention.

## Data Availability

The datasets generated and analysed from this current study can be made available from the corresponding author on reasonable request.

## Appendix 1: activity monitor and sleep time diary

### Please put your activity belt on EVERY day for the next 7 days

The activity belt should be worn 24/7 apart from during water-based activities such as swimming, showering, bathing etc. It should be worn snuggly, on the right hip, under or over clothes. The black button should be on top, so that the word Actigraph is the correct way up.

**Please ensure to record bed and wake times for the days the monitor was worn. Please also record when the belt was taken off (see example below).**

**Table:**

Day	Date	Bedtime	Wake time	Monitor off (reason)	Screen time in minutes per day
Example	26/04/2022	19:30	07:00	18:30–19:00 (bath)	45
1					
2					
3					
4					
5					
6					
7					
	Return the belt and this diary via the pre-paid envelope. Thank you!				

If you have any questions, please do get in touch with [Lead researcher] via e-mail or telephone.

## Abbreviations

WHO: World Health Organisation
ARC: Asynchronous Remote Communities
MVPA: Moderate-to-Vigorous-Physical-Activity
SST: Sedentary Screen Time
SIMD: Scottish Index of Multiple Deprivation
